# Optical Fiber Sensors for Aircraft Structural Health Monitoring

**DOI:** 10.3390/s150715494

**Published:** 2015-06-30

**Authors:** Iker García, Joseba Zubia, Gaizka Durana, Gotzon Aldabaldetreku, María Asunción Illarramendi, Joel Villatoro

**Affiliations:** 1 Department of Communications Engineering, E.T.S.I. of Bilbao, University of the Basque Country UPV/EHU, Alda. Urquijo s/n Bilbao 48013, Spain; E-Mails: joseba.zubia@ehu.eus (J.Z.); gaizka.durana@ehu.eus (G.D.); gotzon.aldabaldetreku@ehu.eus (G.A.); agustinjoel.villatoro@ehu.eus (J.V.); 2 Department of Applied Physics I, E.T.S.I. of Bilbao, University of the Basque Country UPV/EHU, Alda. Urquijo s/n Bilbao 48013, Spain; E-Mail: ma.illarramendi@ehu.eus; 3 IKERBASQUE—Basque Foundation for Science, E-48011 Bilbao, Spain

**Keywords:** structural health monitoring, optical fiber sensors, fiber Bragg grating, long period grating, turbine condition monitoring

## Abstract

Aircraft structures require periodic and scheduled inspection and maintenance operations due to their special operating conditions and the principles of design employed to develop them. Therefore, structural health monitoring has a great potential to reduce the costs related to these operations. Optical fiber sensors applied to the monitoring of aircraft structures provide some advantages over traditional sensors. Several practical applications for structures and engines we have been working on are reported in this article. Fiber Bragg gratings have been analyzed in detail, because they have proved to constitute the most promising technology in this field, and two different alternatives for strain measurements are also described. With regard to engine condition evaluation, we present some results obtained with a reflected intensity-modulated optical fiber sensor for tip clearance and tip timing measurements in a turbine assembled in a wind tunnel.

## 1. Introduction

All kinds of engineering infrastructures undergo aging, and damage appears as a consequence of the loads applied to them, so inspection and maintenance actions are required to predict and lengthen their lifetime thus avoiding catastrophic failures. Among these infrastructures, those related to aviation demand the highest levels of damage detection, since most of them are designed according to a damage-tolerant principle [1]. In order to get lighter structures, these are designed to withstand damages with certain characteristics. Nevertheless, damage-tolerant design involves a considerable amount of time and effort for structure inspection, because there are a wide variety of factors that can cause damage in aircraft structures, most of which are summarized in [2]. Due to the harsh conditions suffered by aircraft structures, periodic and scheduled inspection and maintenance tasks are essential for safe and efficient operation. The cost for the personnel needed to carry out these procedures is high, but the cost due to aircraft downtime during these time-consuming inspections is significantly greater [3]. Hence, automation of the inspection process is a point of capital importance to reduce inspection efforts. In this context, a structural health monitoring (SHM) system can be defined as a set of devices that provides information that allows us to locate, evaluate and predict the loading and damage conditions of a structure. SHM of aircraft structures can perform real time inspection, reducing costs and improving the reliability and performance of these structures [4]. The successful application of SHM to aircraft requires two conditions: it must provide reliable and accurate information about the condition of the structure, increasing the security of critical components and avoiding disasters, and it must be a profitable process for the airline operators by reducing economic losses caused by unproductive downtimes. Therefore, there is a great interest from industry and academia in developing SHM systems that meet both conditions. A wide range of potential SHM technologies is being developed to fulfill these conditions [1], and the most promising options are: electrical strain gauges and crack wires, acoustic emission methods, optical-based technologies, comparative vacuum monitoring [5] and MEMS.

Their intrinsic capabilities, such as insensitivity to electromagnetic radiation, light weight, small size, great sensitivity and resolution, and, above all, their suitability to be embedded into structures [6], make optical fiber sensors (OFSs) very appropriate to perform SHM [7]. There are three possible approaches in order to deploy a SHM system based on optical sensors [8]. The first one uses single-point sensors [9], which is the way in which most of the pressure or temperature sensors operate. The second one employs distributed sensing [10], where the measurand can be obtained at any point of an optical fiber. Finally, there are quasi-distributed systems that use a number of single-point sensors, allowing the sensing of large structures [11]. In this review article we summarize some of the results we have obtained in relation to aircraft SHM projects during the last few years. In Section 2.1, the basic principles of fiber Bragg gratings (FBG) and a quasi-distributed system for impact detection are presented. In Section 2.2, we describe the measurement of strain along a helicopter tail boom by using an elongation sensor, and also the determination of strain in a steel plate by means of a long period grating (LPG). Whereas the LPG is a single-point sensor, the elongation sensor is a distributed one. The performances of both sensors are compared with the results obtained using FBGs. Finally, in Section 3 the design and results of an optical sensor that has been used to carry out the SHM of an aircraft turbine are reported.

## 2. SHM in Aircraft Structures

OFSs for monitoring the strain in aircraft structures can be classified into the following categories [12]: intensity-based OFSs [13], interferometric OFSs [14], grating-based sensors and distributed OFSs [15]. Intensity-based OFSs were the first optical sensors employed to measure the strain, and they are the simplest and least expensive optical sensors for SHM. Interferometers work on the principle of the interference caused by a reflected monocromatic light with its original source. Several interferometer configurations have been proposed, being Fabry-Perot the most widely utilized. There are different techniques for implementing a distributed OFS; optical time domain reflectometry (OTDR) based on Rayleigh scattering, Raman optical time domain reflectometry (ROTDR) or Brillouin optical time domain reflectometry (BOTDR), based on Raman and Brillouin scattering, respectively. BOTDR resolution and acquisition time has been improved by Brillouin optical time domain analysis (BOTDA) using the phenomenon know as Stimulated Brillouin Scatter. Among grating-based sensors, FBGs are probably the most mature and widely employed optical sensors for SHM of engineering structures, due to their fast development achieved in recent years [16,17]. For the particular case of aircraft structures, even though FBGs have demonstrated to be a promising technology to monitor strain [18], strain gauges still remain being the most used method to perform strain measurements in operational aircraft structures. FBGs have important advantages over conventional strain sensors, namely:

(a) Intrinsic benefits of optical fiber sensors such as light weight and small size, absence of electromagnetic interference, high sensitivity and resolution, *etc.* [19].
(b) Suitability for being attached to a structure or embedded in composite materials [20].
(c) Wavelength-encoded sensing in a way that is totally independent of the optical intensity [21], which confers them long-term stability without the need of recalibrating.
(d) High multiplexing capability: since each FBG has a narrow-wavelength operating band, it is possible to multiplex several sensors in the same fiber, thus allowing for simultaneous multi-point measurements [22].
(e) Different magnitudes can be measured using FBGs, such as strain, temperature, vibration or humidity.

Regarding strain measurement, FBGs show a similar behavior to strain gauges. However, even though strain gauges are robust, low cost and well known, they present important disadvantages such as their long-term signal drift or time-consuming and complex wiring [23], since two leads per sensor are required. The multiplexing capability of the FBGs overcomes the complex wiring drawback. This characteristic has been reported in several works [11,24], in which the multiplexing capability of the FBGs provides a size optimization for the practical implementation of a quasi-distributed SHM system. The additional possibility of being embedded in the structures allows the use of FBGs to identify damage in composite structures [20], as well as the monitoring of composite cure during its manufacture [25].

### 2.1. Fiber Bragg Gratings

**Figure 1:**
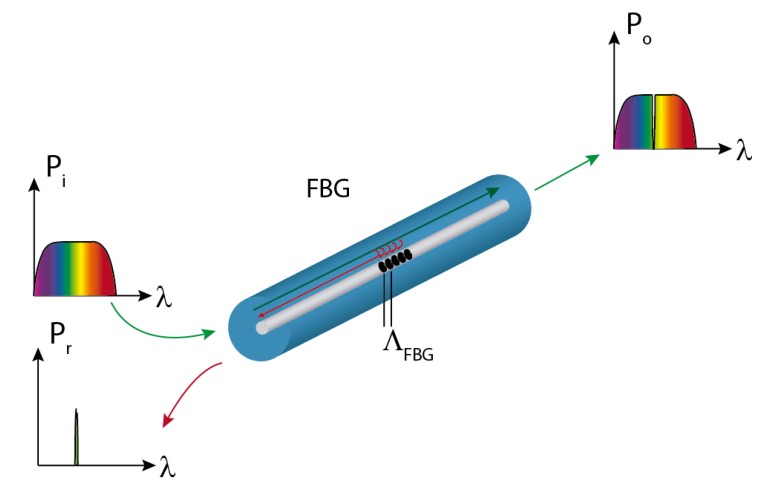
FBG concept and working principle.

An optical fiber grating can be defined as a periodic perturbation pattern in the refractive index of the fiber core, in such a way that certain wavelengths of the guided mode are diffracted either into other radiation modes or into cladding modes [26]. In the former case, the device is known as a short-period fiber grating or fiber Bragg grating. Such gratings have a sub-micron period and a total length ranging from 1 mm to 10 mm. A Bragg grating allows most of the power (P_o_) to propagate forward. However, when the guided mode in a single-mode fiber reaches the grating, a certain portion of its incident power (P_i_) is reflected at each grating plane. The electric fields are added up only if the Bragg condition given by Equation (1) is satisfied:

(1) $$\lambda_{B}=2\cdot n_{eff}\cdot \Lambda$$

where λ*_B_* is the resonant wavelength or Bragg wavelength, *n_eff_* is the effective index of the mode and Λ is the grating period. The working principle of the FBGs is illustrated in Figure 1.

There are two basic techniques to inscribe Bragg gratings in fibers. The first one uses ultraviolet light to change the refractive index of photosensitized fibers. The photosensitivity process typically consists in doping the fiber core with germanium, so that the fiber becomes receptive to UV-induced refractive index changes. Several methods have been employed to enhance the photosensitivity, such as high temperature treatment of hydrogen preforms, B-Ge co-doping, flame brushing, and high-pressure hydrogen loading of fibers [27]. The second fabrication technique makes use of a femtosecond laser to write a grating in non-photosensitive single-mode commercial fibers [28]. The main advantages of this technique are the shortening of the processing time, the reduced requirements for the fabrication process and the mechanical strength of the gratings. Currently, the most employed methods to inscribe the gratings are holographic techniques [29] and phased mask methods [30]. The point-by-point technique is usually employed when femtosecond lasers are used to inscribe the gratings [31].

As mentioned before, the measurand is encoded in the variations of the Bragg wavelength. Any change in the temperature or in the strain of the fiber causes a change in the grating period and/or in the effective index, shifting the central wavelength reflected by the grating. When the FBG is subjected to a tension, the change in the Bragg wavelength is given by Equation (2):

(2) $$\frac{\Delta \lambda_{B}}{\lambda_{B}}=(1-P_{e})\cdot \Delta \varepsilon$$

where ε is the longitudinal strain in the FBG and *P_e_* is the effective photo-elastic constant of the fiber core material. In Equation (2), *P_e_* can be calculated as:

(3) $$P_{e}=\frac{n_{eff}}{2}\left[p_{12}-\upsilon \left(p_{11}+p_{12}\right)\right]$$

where *p*_11_ and *p*_12_ are the elasto-optic coefficients, which represent the change in the refractive index due to the applied strain, and υ is the Poison ratio. The temperature changes affect the Bragg wavelength in two ways. On the one hand, the expansion coefficient of the fiber material (α) characterizes the thermal expansion of the grating, and, on the other, the variations in the refractive index are determined by the thermo-optic coefficient (ζ). Therefore, the change in the Bragg wavelength with temperature can be expressed as:

(4) $$\frac{\Delta \lambda_{B}}{\lambda_{B}}=(\alpha +\zeta )\cdot \Delta T$$

Combining Equations (2) and (4) we get that the total Bragg-wavelength variation can be calculated as:

(5) $$\frac{\Delta \lambda_{B}}{\lambda_{B}}=(\alpha +\zeta )\cdot \Delta T+(1-P_{e})\cdot \Delta \varepsilon$$

**Figure 2:**
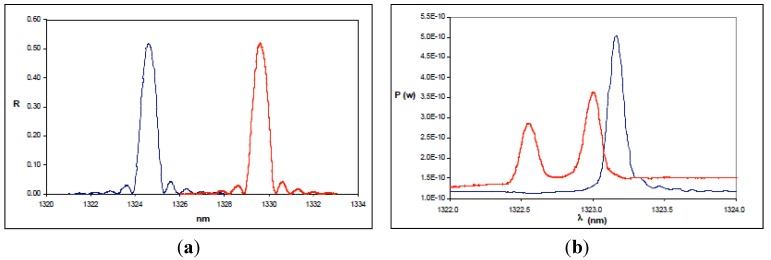
(**a**) Ideal behavior of the reflected spectrum of an FBG subjected to a longitudinal strain; (**b**) Effect of a transverse stress on the reflected spectrum of the FBG. In all figures the blue line represents the non-perturbed spectrum of the FBG and the red one is the spectrum after the application of the load.

Thus, it is clearly seen that the Bragg wavelength presents a cross-sensitivity phenomenon (it is sensitive to both temperature and strain). For strain measurements, the effect of temperature has to be compensated. Different configurations have been reported for this purpose [32,33,34,35,36,37,38,39,40,41,42,43,44]. However, when temperature gradients in the structure are not excessive, the use of a single FBG insulated from the effect of the strain is enough to compensate the effect of temperature in the rest of the FBGs, thus allowing us to discriminate the variations in the Bragg wavelength that appear as a consequence of strain. FBGs are excellent strain sensors when the load is applied in the axial direction of the sensor. In this situation, the FBG only undergoes contraction or elongation. If there is a transverse stress applied, the fiber presents birefringence due to the variation experienced by the effective refraction index on each propagation axis [45]. As a consequence, the grating exhibits two different Bragg conditions, and the approximately Gaussian-shaped reflected spectrum of the FBG splits into two peaks, as represented in Figure 2 [46]. Another similar phenomenon appears when there is a non-uniform strain field, because, in this case, the spectrum is broadened and even split into several peaks [20], which makes it difficult to track the Bragg wavelength. Both problems can arise when the FBG is embedded in composite materials. In conclusion, FBGs constitute a mature technology and compared to strain gauges, provide multiple benefits and attractive properties, such as multiplexing capability, the possibility of being embedded in the structure, long term stability and a competitive cost per channel. Nevertheless, some issues, like non-uniform or multidirectional complex strain conditions and the lack of aircraft certification must be solved, so that FBGs can be used in SHM systems for aircraft structures.

**Figure 3:**
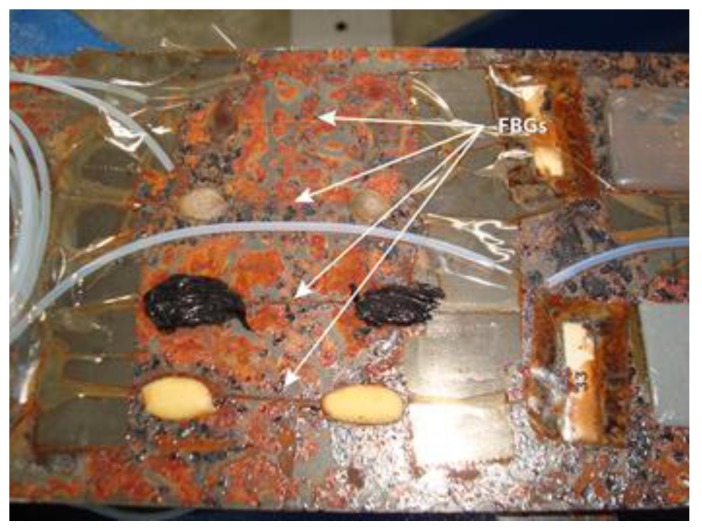
Photograph of four FBGs bonded with different adhesives to the F114 steel specimen after being aged for two months in a climate chamber.

Another important concern is the effect of aging on the response of the FBGs under extreme conditions. In [47] an analysis of the influence of aging FBGs is presented. FBGs were bonded on three specimens using different adhesives to evaluate their performance against aging. In order to reproduce aging under real conditions, two of them were stored in a climate chamber at 70 °C and 90% relative humidity for one and two months, respectively. The damage produced in the specimen aged for two months can be observed in Figure 3.

**Figure 4:**
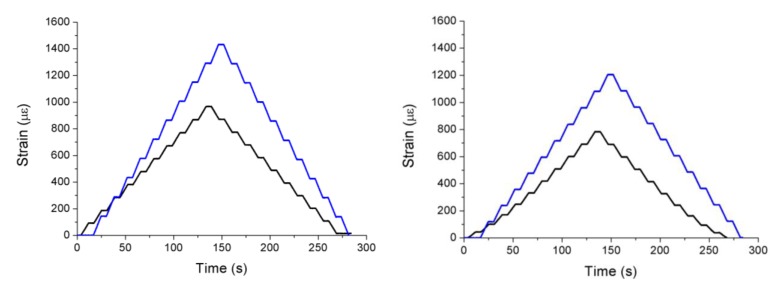
Spectra of two FBGs bonded on the specimen before (**black line**) and after (**blue line**) aging for one month in a climate chamber.

**Figure 5:**
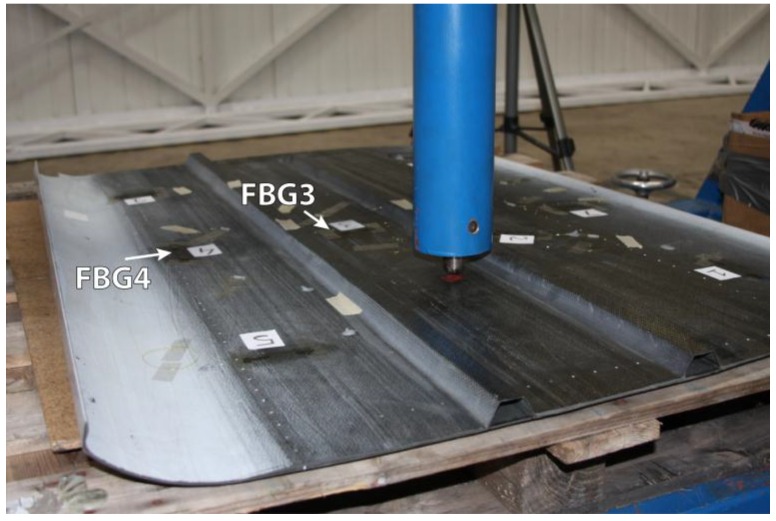
Instrumented specimen for impact detection and certified impact machine used in the tests.

The results of the tests on the specimens previously aged for one and two months are very similar and, unexpectedly, they show that the readings of the FBGs are about 50% higher than those obtained before the aging. Since the maximum working temperature of the FBG was never exceeded, this discrepancy in the readings must be caused by the effect of humidity. Its influence can make the refractive index vary, shifting the Bragg wavelength and yielding much higher strain values than expected, so in order to avoid this problem, it is necessary to properly insulate the FBG from humidity. The results for two FBGs of the specimen aged for one month for 200 kN traction stress tests are displayed in Figure 4. The traction stress was applied in steps of 20 kN. In most cases, FBGs are employed in SHM applications to detect strain or temperature variations in a quasi-static way. However, if FBGs are used to detect impacts in aircraft structures, vibrations at higher frequencies must be analyzed [48]. We developed a proof-of-concept system for impact detection in composites using FBGs [49]. The objective was to obtain the arrival times of the surface mechanical waves produced by an impact in different points of the structure. Using these times, the impact location could be determined by triangulation. Two arrays of five FBGs were instrumented on a piece of Carbon Fiber Reinforced Polymer (CFRP). In order to detect the mechanical waves due to the impacts in as many directions as possible, nine of the FBGs were placed following the arrangement given in [50]. The remaining FBG was insulated from the vibrations and employed to compensate the effect of the temperature. The final layout can be observed in Figure 5. The test procedure included five different impact points; each impact was repeated three times with increasing energies from 0.245 J to 29.4 J.

**Figure 6:**
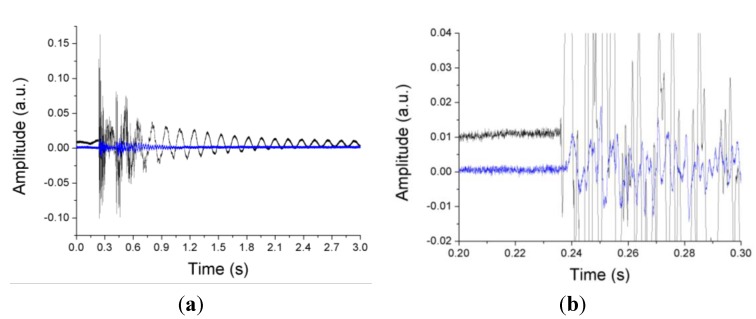
(**a**) Response of two different FBGs (FBG3 and FBG4 shown in Figure 5) to the mechanical waves produced by an impact; (**b**) Detailed view of the arrival of the mechanical waves to the FBGs.

A 20 kHz interrogator was employed to obtain the response of the FBGs and Figure 6 shows the difference between the arrival times of the wavefronts generated by the impact for two FBGs.

### 2.2. Alternatives to Fiber Bragg Gratings

FBGs are probably the most well-known, developed and widely used OFSs for SHM in aeronautical structures. Their characteristics make them the most promising technique to evaluate the condition of these structures, but in order to have a broad vision of the possibilities that OFSs offer, it is necessary to consider different alternatives to FBGs that can provide suitable solutions to specific cases. In this section we present two different sensors as possible alternatives to FBGs. Their performances are analyzed and compared to those of the FBGs working in the same conditions.

#### 2.2.1. Plastic Optical Fiber Elongation Sensor

In this section we present a plastic optical fiber (POF) elongation sensor as a possible alternative to FBGs for the measurement of strain and vibrations in aircraft structures. The sensor’s operating principle relies on the measurement of the phase shift between two sinusoidally-modulated optical signals. These signals travel in two different POFs, one used as reference and the other one bonded to the surface of the aircraft structure under test [51].

**Figure 7:**
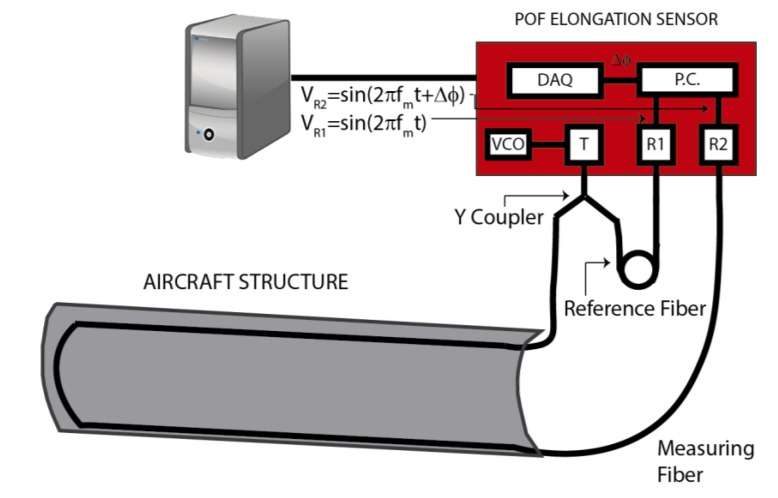
POF elongation sensor components and set-up. VCO: Voltage-controlled oscillator; T: Transmitter; R1: Receiver 1; R2: Receiver 2; P.C.: Phase comparator; DAQ: Data acquisition module.

The main advantage of this sensor is the use of low-cost components. Conventional POFs are used as the sensitive component of the sensor. Due to the multimode nature of these fibers, the phase and the polarization state are not maintained along the fiber, so the only possible transduction technique must be based on intensity modulation [52]. In Figure 7 a schematic representation of the sensor is depicted. The output of the voltage-controlled oscillator (VCO) is a sinusoidal voltage with an adjustable frequency (*f_m_*). This VCO signal is applied to the transmitter, which is basically a light-emitting diode at 650 nm. The Y coupler divides the optical signal in two identical ones that will be transmitted by the reference fiber and the measuring fiber, respectively. The receiver, essentially a photodiode, transforms into voltages the optical signals coming out from the fibers as:

(6) $$V_{R1}=\sin\left(2\pi f_{m}t\right)$$

(7) $$V_{R2}=\sin\left(2\pi f_{m}t+\Delta \phi \right)$$

When the measuring fiber is elongated or shortened due to the strain applied to the aircraft structure, the phase difference between the signals (∆ϕ) given by Equations (6) and (7) changes. In order to capture the phase difference, the outputs of both receivers are connected to a phase comparator from which the elongation of the fiber (∆*l*) can be estimated as [53]:

(8) $$\Delta l=\frac{c}{n_{core}}\cdot \frac{1}{360^{\circ }}\cdot \frac{\Delta \phi }{f_{m}}$$

where *c* is the speed of light in vacuum and *n_core_* is the refractive index of the fiber core. Once the phase comparator signal is acquired by a data acquisition module (NI-USB3210 from National Instruments), a Labview program processes the signal to obtain the elongation of the fiber.

**Figure 8:**
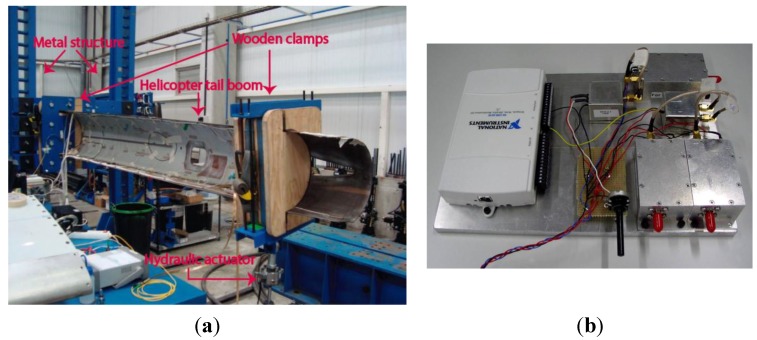
(**a**) Experimental set-up for the tests performed on the tail boom; (**b**) Prototype of the electronics for the POF elongation sensor.

In contrast to the discrete-point measurement obtained using FBGs, the POF elongation sensor provides a distributed measurement of the strain in the structure, since the overall fiber elongation is taken into account. In previous works [54,55], the sensor was employed to characterize the response of a wing flap to flexural-type loading conditions. We compared the results of the POF elongation sensor to those provided by FBGs. For these tests, the structure to be assessed was a helicopter (Eurocopter EC 135) tail boom which can be observed in Figure 8. The assembly is composed of a rigid metal structure on one end of the tail boom and a hydraulic actuator on the other one. The load applied by the actuator produces bending of the tail boom and, therefore, stress in the structure. Three different fatigue cycles were applied to the tail boom and each load cycle lasted for 16 s. The forces applied in the fatigue cycles were 0.25, 0.50 and 0.75 kN.

Besides the POF elongation sensor, ten FBGs in two different fibers were instrumented on the surface of the tail boom for the sake of comparison. In the first fiber, four FBGs were installed in the longitudinal direction of the tail boom and the last one in a perpendicular direction. For the second fiber, four FBGs were placed longitudinally and one was used for temperature compensation. The response of the FBGs was measured using the SM130-200 interrogator from MicronOptics.

**Figure 9:**
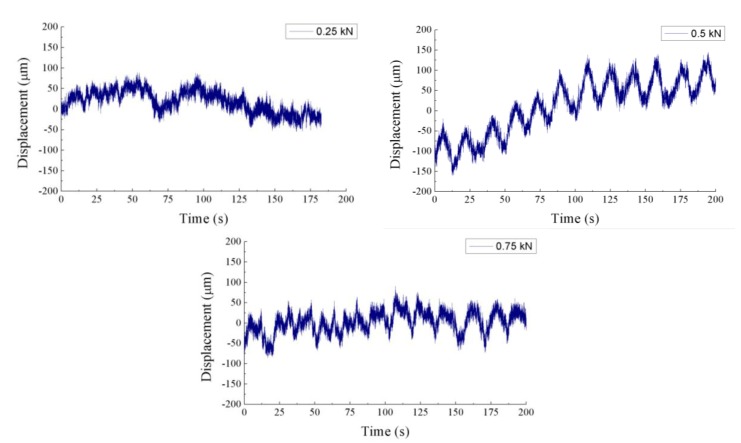
POF elongation corresponding to the three fatigue cycles of 0.25 kN, 0.5 kN and 0.75 kN.

**Figure 10:**
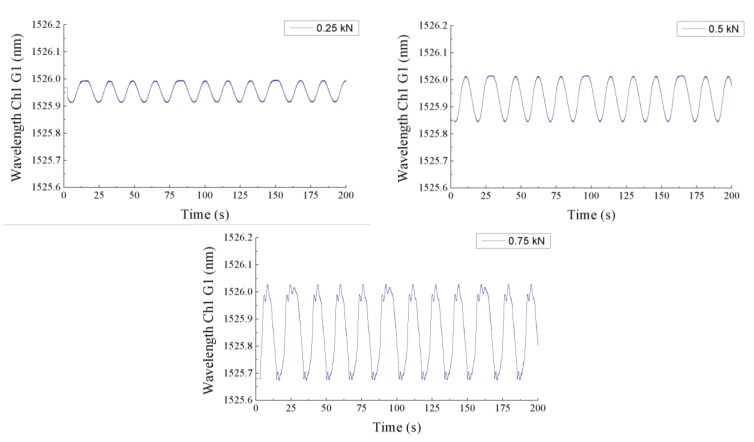
Wavelength variations due to the strain in the tail boom provided by the first FBG during the three fatigue cycles.

The results obtained by the POF elongation sensor for several load cycles can be observed in Figure 9. When a force of 0.75 kN or 0.5 kN is applied, the cycles are clearly detected by the sensor. However, for the case of 0.25 kN the noise makes it impossible to distinguish the stress cycles in the post-processed signal. The goal of these tests was to demonstrate the capability of the POF elongation sensor to detect the load cycles on the tail boom. Although this goal was achieved, more tests should be carried out to ensure the reliability of the sensor, and the quality of the output signal must be improved. The offset variations in the displacement signals are due to the drift of the modulation frequency provided by the VCO, which is interpreted by the phase comparator as a change in the phase of the received signals. Hence, the VCO performance must be optimized to stabilize the modulation frequency and avoid changes in the offset of the output signal. Another way to improve the POF elongation sensor would be to bond the reference fiber on the outer surface of the tail boom, so that the phase difference between the two optical signals would increase. Thus, increasing the sensitivity of the sensor. For the sake of comparison, the results obtained for one of the FBGs are plotted on Figure 10. The signals are obviously less noisy than those of the POF elongation sensor. The strain in the structure is represented by the changes of the reflected wavelength in the FBG for the three applied forces. As expected, the strain given by the FBG placed in the perpendicular direction of the tail boom is noticeably smaller than the ones provided by the FBG placed longitudinally.

The FBGs are much more sensitive than the POF elongation sensor and offer an excellent performance for a single-point measurement. On the other hand, the POF elongation sensor provides a distributed measurement of the strain in the whole structure and it is a cost-effective option when the required measurement resolution is not so high.

#### 2.2.2. Long Period Gratings in Microstructured POF

**Figure 11:**
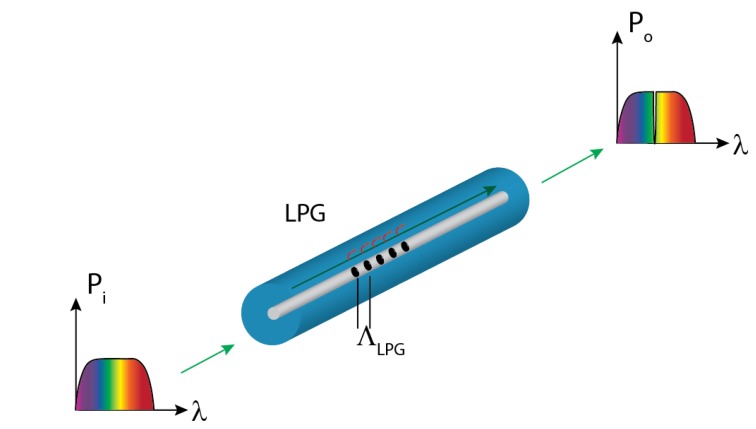
LPG concept and working principle.

In this section we present the use of long period gratings in microstructured POFs (mPOFs) as an option to measure strain in aircraft structures instead of FBGs. mPOFs are the counterpart version of the photonic crystal fibers using polymer fibers instead of glass fibers. In the mPOFs a pattern of air channels along the POF guides the light and confers unique properties to the fiber [56]. Among these properties, the single-mode and polarization-maintaining behavior, combined with the intrinsic flexibility and ease of connection of the POF are particularly interesting for sensing purposes. In order to obtain an LPG in an mPOF, a periodic modification of the cladding structure of the mPOF is written along the direction of light propagation [57], so that the core mode is coupled to cladding modes, which are rapidly attenuated, resulting in depressions in the transmission spectrum at wavelengths where the Equation (9) is satisfied [58]:

(9) $$m\lambda =(n_{core}(\lambda )-n_{cl}(\lambda ))\Lambda_{LPG}$$

where *m* is the order of the interaction, *n_core_* and *n_cl_* are the effective indices of the core and the cladding, and Λ*_LPG_* is the period of the grating. Unlike FBGs, this period is much larger than the wavelength of the light propagating along the mPOF, so LPGs are easier to fabricate. In the case of LPGs, the length of the structure is in the order of 30 mm, whereas the grating period range is typically from 100 µm to 1 mm [59]. The LPG causes the coupling between the core-guided mode and the forward-propagating cladding modes at certain wavelengths, which causes “notches” in its transmission spectrum. The working principle of the LPG is depicted in Figure 11.

**Figure 12:**
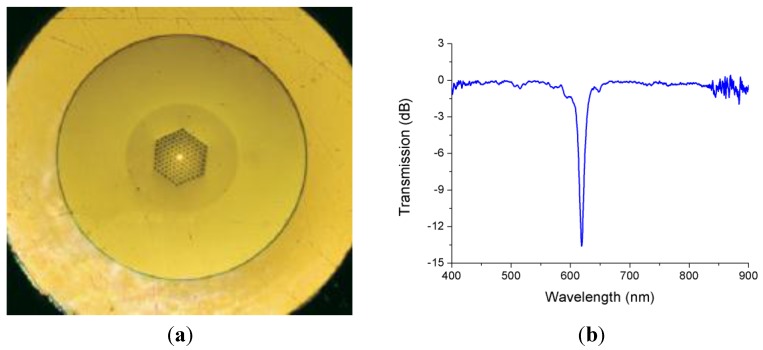
(**a**) Cross section of the mPOF used to inscribe an LPG; (**b**) Normalized transmission spectrum of the LPG used in the tests.

Figure 12 shows the mPOF employed to inscribe the LPG that we used in the tests and its normalized transmission spectrum. Since changes in the strain of the mPOF can modify the period of the LPG, the wavelength that matches the phase condition changes. As a result, the depression at 620 nm in the spectrum is sensitive to strain and its wavelength changes accordingly.

**Figure 13:**
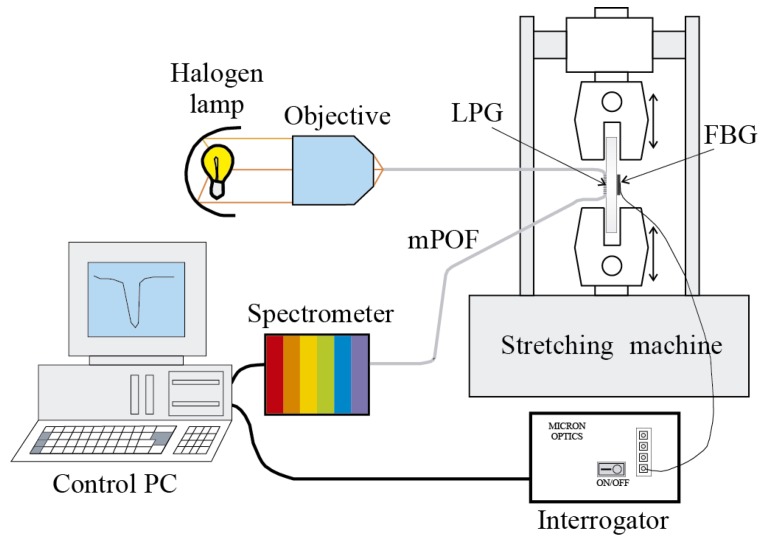
Experimental set-up for the traction-compression tests in a steel plate specimen.

In order to detect the changes of the resonant wavelength in the LPG, light from a white source is launched into the fiber and the spectrum of the output light is obtained by using the USB4000 miniature fiber optic spectrometer from Ocean Optics. The experimental set-up is represented in Figure 13 [60].

**Figure 14:**
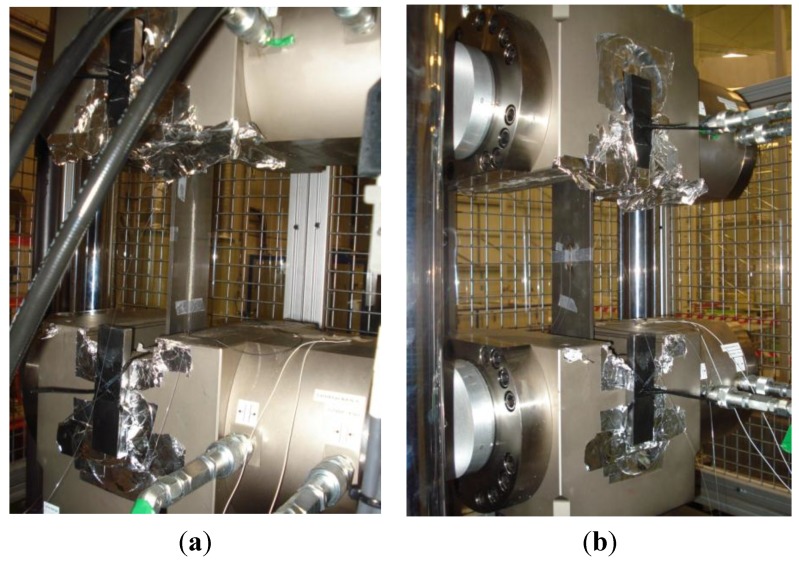
(**a**) Plate specimen assembled on the stretching machine with the LPG; (**b**) The FBG instrumented on the other side of the plate.

The LPG mPOF with a total grating length of 60 mm was bonded to the surface of a one-meter-length steel plate, whereas on the other surface of the plate an FBG was instrumented as a reference sensor (see Figure 14). The SM130-200 interrogator was employed to capture the response of the FBG. The plate was subjected to a triangular-like tension cycle at a frequency of 0.2 Hz by means of a servo-hydraulic actuator.

**Figure 15:**
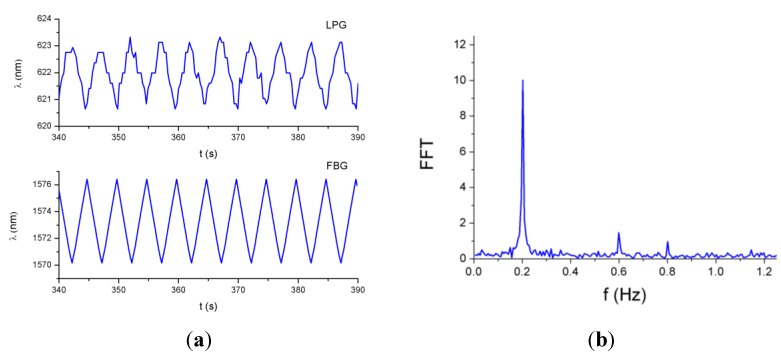
(**a**) Responses of the LPG and the FBG to a triangular-like tension cycle at a frequency of 0.2 Hz; (**b**) FFT of the LPG response.

As can be observed in Figure 15a, the responses of the sensors are 180° out of phase because of the opposite variation of the wavelength with respect to the strain for FBGs and LPGs. In the case of the LPG, the fluctuations in the signal are due to the resolution of the spectrometer employed in these tests. To smooth these fluctuations a spectrometer with higher resolution can be used, or low-pass filtering can be applied to the signal. The spectrum corresponding to the LPG signal is represented in Figure 15b; the peak corresponding to the fundamental frequency (0.2 Hz) is clearly identified.

## 3. SHM in Aircraft Engines

In addition to monitoring the integrity of the aircraft structures it is also essential to ensure the condition of the engines. Optical methods can be employed to monitor different engine parameters such as vibration, temperature, pressure or rotational speed of the engine [61]. They are commonly based on FBGs or interferometric technology but we have developed a reflective intensity-modulated method to assess the performance and SHM of aircraft engines. Several prototypes of this optical sensor are being successfully employed to characterize turbines of aircraft engines in a wind tunnel installation. Other systems based on microwave [62], eddy current [63], or capacitive [64] probes have been developed and in [65] are compared to different optical techniques.

**Figure 16:**
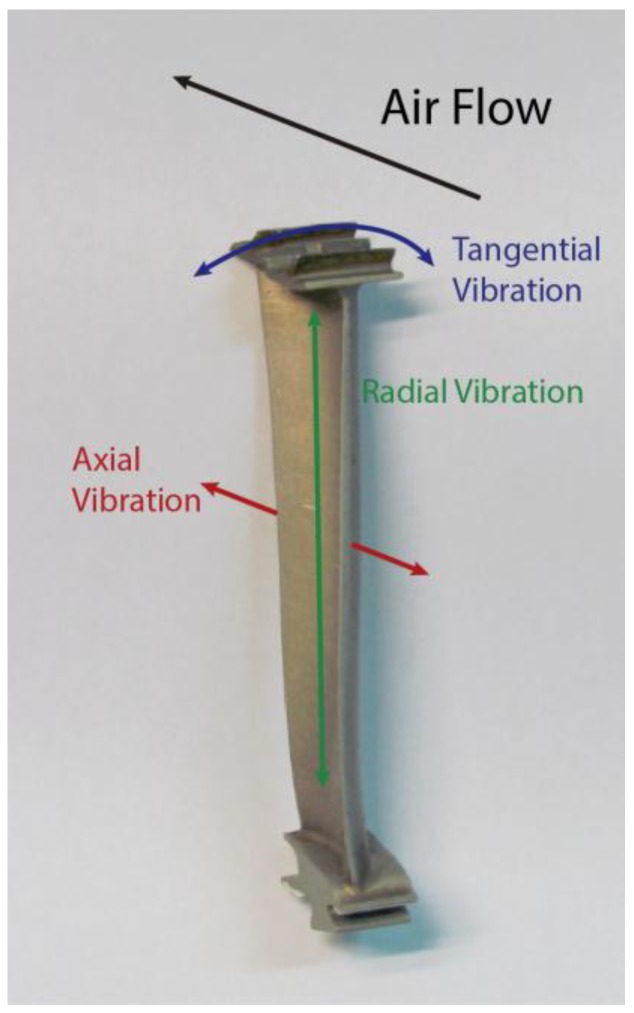
Possible vibrations of the blade during turbine operation: radial (green), axial (red) and tangential (blue). The turbine blade is courtesy of the Aeronautical Technologies Center.

There are three different types of blade vibrations that can be analyzed, namely, radial, tangential and axial (see Figure 16). Currently, the sensor is being used to quantify the radial and tangential vibrations.

The distance between the tip of the blades of an aircraft engine and its casing is known as tip clearance (TC). The measurement of this distance is equivalent to evaluating the radial vibrations of the blades. This measurement is interesting since it is possible to get a more efficient engine by reducing its TC [66]. Several kinds of sensors, such as capacitive or discharge probe sensors, can be used to carry out these measurements. In Section 3.1 we compile the improvements in the design of an optical sensor for TC measurements of turbine rigs and present another practical application of this optical sensor.

Tip timing is a well-known [67] technique to compute the amplitude of blade vibrations using the differences between the theoretical and the real arrival times of the blades. Since our sensor is placed in a radial position, the tangential vibrations of the turbine blades are also assessed. These vibration amplitudes provide useful information about the turbine operation and the SHM of the blades, since it is possible to detect cracks in the blades or flutter. In Section 3.2 we present a set of tip timing tests for a turbine with 146 blades.

### 3.1. Tip Clearance Measurements

**Figure 17:**
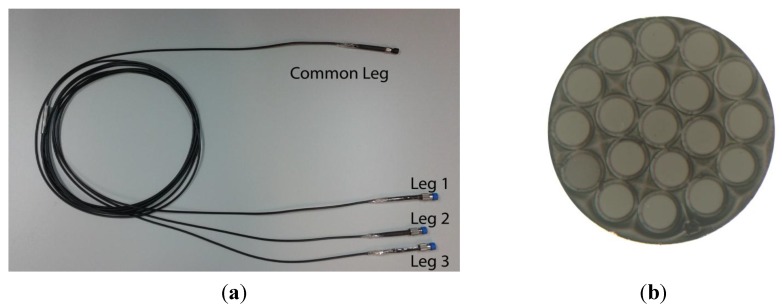
(**a**) Trifurcated bundle of optical fibers; (**b**) Microscope view of the cross section of the bundle common leg.

The main component of the optical sensor is a trifurcated bundle of optical fibers with a common leg on one side and three legs on the other. In Figure 17, the bundle and a cross section view of the common leg are depicted.

**Figure 18:**
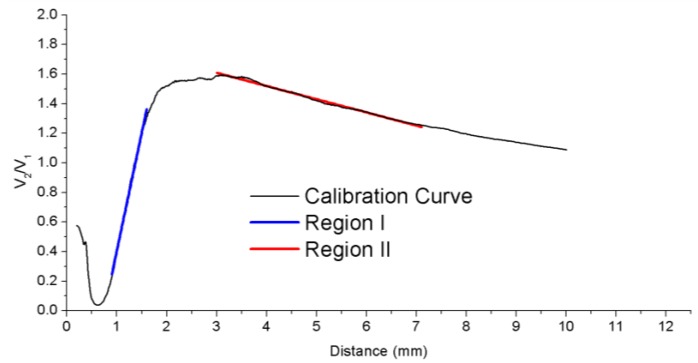
Calibration curve of the sensor showing the two possible regions of measurement: region I (front-slope) in blue and region II (back-slope) in red.

The central fiber guides the light from a laser module connected at leg 1 to the probe end, so that the blades of the turbine are illuminated. This fiber is surrounded by two rings of receiving fibers which collect the reflected light from the blades. The set of receiving fibers corresponding to the first ring (6 fibers) and the second ring (12 fibers) are gathered in bundle legs 2 and 3, respectively. At the end of these legs two photodetectors are connected to convert the optical signals into voltage. The distance from the sensor tip to the blade is calculated from the quotient of these voltages to minimize the effects of fluctuations of the light source or variations in the reflectivity of the surface [68,69]. Detailed information about the configuration of the sensor and the set-up to perform the measurements is provided in [70]. The calibration curve of the sensor is depicted in Figure 18. Previous works carried out the measurements using the front-slope region (region I), whereas our sensor operates in the back-slope region (region II). In this region, there is a trade-off between the sensitivity of the sensor and its linear range, so that the TC can be measured for larger distances but with lower sensitivity.

Several configurations have been tested to improve the sensitivity of the sensor. The two main changes have been the reduction of the modal noise at the output of the optical probe and the employment of asymmetric gain for the photodetectors [71]. The evolution of the sensor is summarized in Table 1. Although the third configuration shows the best precision, its maximum operating temperature is 60 °C, which restricts the measurements to low temperatures. In contrast, the bundles of glass fibers can work at temperatures up to 350 °C. Therefore, we decided to use the fourth configuration for future measurements.

**Figure 19:**
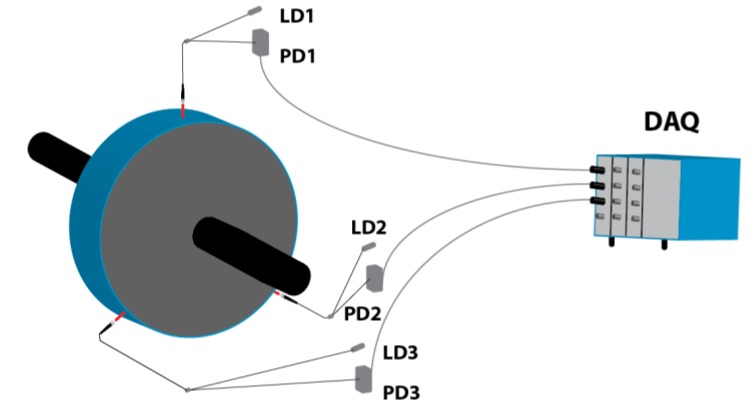
Set-up of the three sensors to evaluate the vibrations in the rotating disk. LDx: Laser diode x; PDx: Photodetector x; DAQ: Data acquisition system.

The sensor can be used for other purposes beyond the measurement of the TC in the turbines. For instance, three sensors have been used to assess the vibrational behavior of a rotating disk that separates two chambers of different pressures in a real aircraft engine. This disk fluttered under certain circumstances and three sensors were installed to detect these vibrations during some tests carried out in a wind tunnel. The sensors were placed at 120° with respect to each other, as shown in Figure 19. The three ends of the probes were placed 4.8 mm away from the rotating disk.

**Figure 20:**
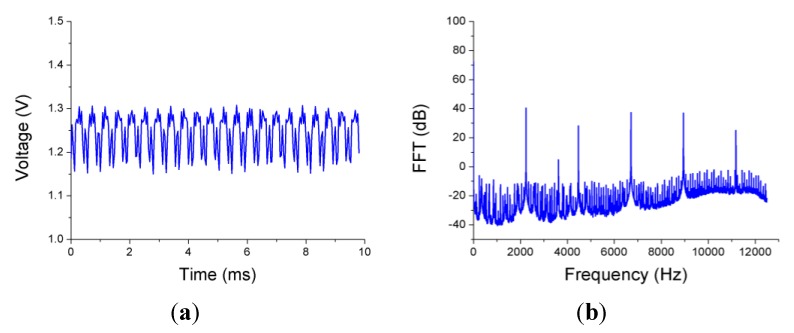
(**a**) Voltage output from one of the sensors when the disk is static and the pressure difference is 2.75 bar; (**b**) FFT of the signal.

The disk vibrated even when it was not rotating due to the air flow between the edges of the disk and the casing, so measurements for several rotational speeds from 0 to 3000 revolutions per minute (rpm) and multiple pressure differences at both sides of the disk were carried out. In Figure 20 the signal obtained from one of the sensors and its FFT when the disk is static with a pressure difference of 2.75 bar are shown. The FFT presents a clear peak at 2236 Hz that indicates the frequency of the radial vibration of the disk.

**Table 1:** Characteristics of the different configurations of the TC sensor.

Config.	1st Sensor Configuration	2nd Sensor Configuration	3rd Sensor Configuration	4th Sensor Configuration
**Light source (Laser)**	7 mW 650 nm	30 mW 650 nm	30 mW 650 nm	20 mW 650 nm
**Optical fibers bundle**	Multimode Ø*_core_* = 100 µm NA = 0.22	Scrambler + Multimode Ø*_core_* = 100 µm NA = 0.22	POF Ø*_core_* = 240 µm NA = 0.5	Single-mode illuminating fiber Ø*_core_* = 4.3 µm NA = 0.12 Multimode receiving fibers Ø*_core_* = 100 µm NA = 0.22
**Photodetectors gain**	Symmetric gain G_1_ = G_2_ = 0.75 × 10^4^ V/A	Asymmetric gain G_1_ = 0.75 × 10^5^ V/A G_2_ = 2.38 × 10^5^ V/A	Asymmetric gain G_1_ = 0.75 × 10^5^ V/A G_2_ = 2.38 × 10^5^ V/A	Asymmetric gain G_1_ = 0.75 × 10^5^ V/A G_2_ = 2.38 × 10^5^ V/A
**Calibration curve**	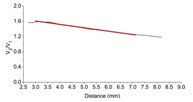 V_2_/V_1_ = −0.089d + 1.8783	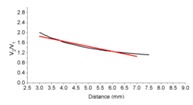 V_2_/V_1_ = −0.2002d + 2.4578	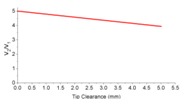 V_2_/V_1_ = −0.213d + 5.0064	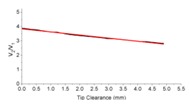 V_2_/V_1_ = −0.2167d + 3.8448
**Cross section of the common leg**	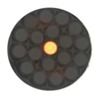	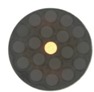	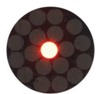	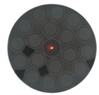
**Laboratory precision**	141 µm	51 µm	33 µm	24 µm
**Wind tunnel precision**	24 µm	-	25 µm	28 µm

**Figure 21:**
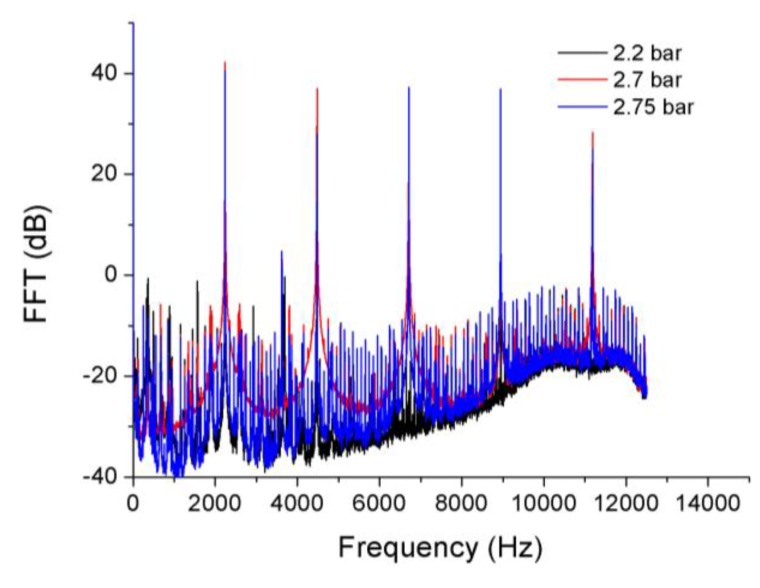
FFTs of the sensor signal obtained at pressure differences of 2.2, 2.7 and 2.75 bar.

Figure 21 shows how the amplitude of the vibration changes when the pressure difference varies. The maximum amplitude of the vibrations is achieved at a pressure difference of 2.7 bar. In Table 2 the amplitude, expressed in dB with respect to one micrometer, and the frequency of the disk vibration for all measured pressure differences are listed. The uncertainty in the frequency measurements is 2 Hz, it could be improved employing a higher number of points to perform the FFT. The obtained frequency values match exactly with those given by one strain gauge instrumented on the surface of the disk.

**Table 2:** Amplitude and frequency of the disk vibrations at several pressure differences when the disk is static.

Pressure Difference (bar)	Amplitude (dB)	Frequency (Hz)
**2.2**	14.3	2238
**2.3**	19.5	2238
**2.4**	31.6	2238
**2.5**	38.5	2238
**2.6**	32	2236
**2.7**	42.2	2236
**2.75**	40.5	2236

### 3.2. Tip Timing Measurements

**Figure 22:**
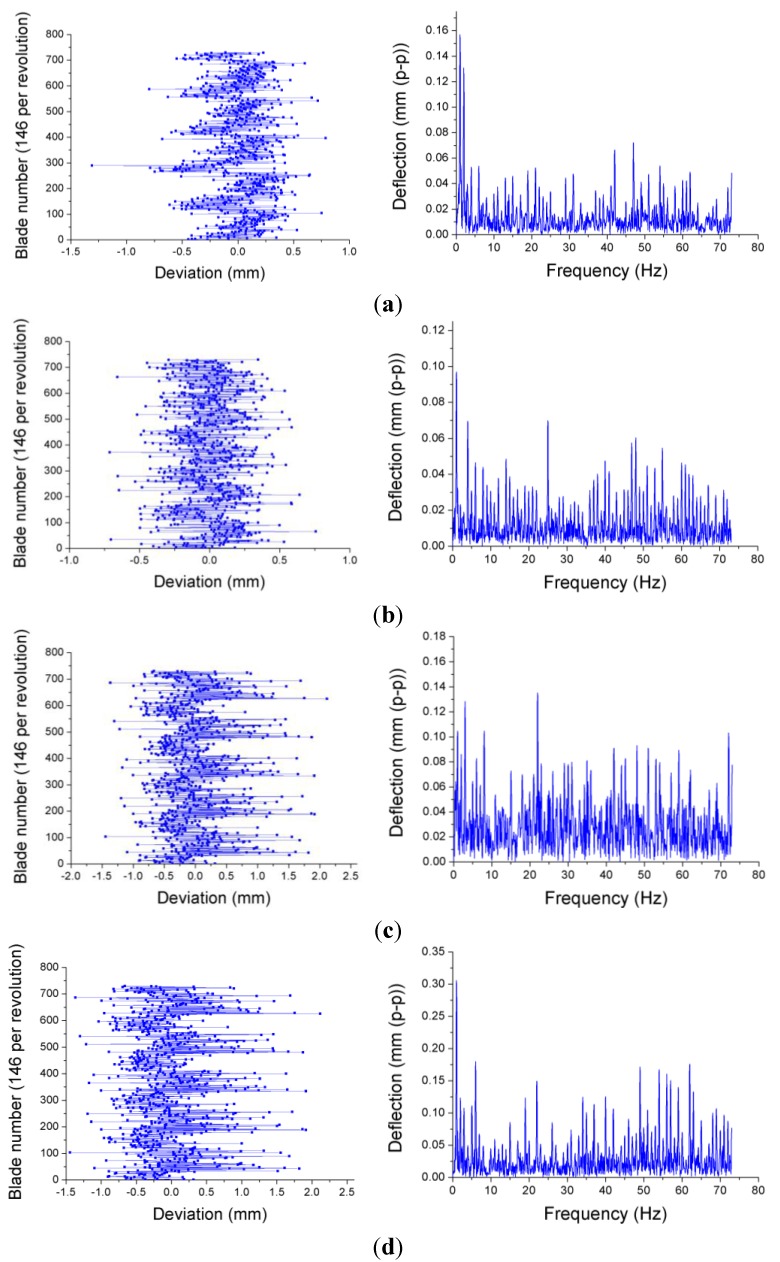
Deviations of each blade form the equilibrium position and travelling wave spectrum of a 146-blade turbine working at 2422 rpm (**a**); 3390 rpm (**b**); 4360 rpm (**c**) and 5570 rpm (**d**).

As we mentioned before, tip timing is a technique used for the evaluation of the amplitude of tangential vibrations of the blades. Taking into account the difference between the theoretical and the real blade arrival times, the amplitude of the blade deflection is obtained. The entire signal processing to get the deflection of each blade is extensively explained in [70]. The travelling wave spectrum is calculated as the FFT of the deflection values for the blades, and represents the average amplitude of the vibration of the blades at a certain nodal diameter. Figure 22 depicts the deviations of every blade of a 146-blade turbine turning at different rotational speeds. These deviations provide useful information to monitor the turbine blades, since they allow the detection of cracks or flutter when the amplitude of the blade vibration exceeds a predefined alarm value.

**Figure 23:**
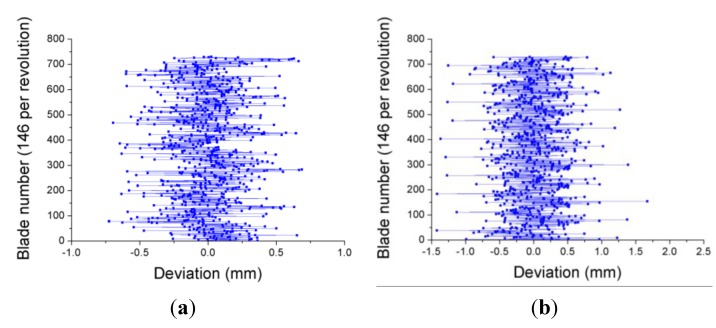
Deviation of each blade form the equilibrium position obtained four days later at 3390 rpm (**a**) and 5570 rpm (**b**).

Although there are some exceptions, the results show a trend towards increasing the amplitude of the blade vibrations as the rotational speed gets higher. The tests were repeated again four days later. In Figure 23 the results obtained at 3390 and 5570 rpm in this second test campaign are shown. As can be observed, the amplitudes of the vibrations are quite similar for both cases. This fact indicates that the blades of the turbine did not suffer any significant damage between both tests.

## 4. Conclusions

Aircraft structures, due to their operating conditions and design principles, require intense inspection and maintenance operations. SHM based on optical sensors could play an important role in these operations provided that two issues can be overcome. The potential economic and life-safety benefits of optical sensors for SHM should be clearly demonstrated to the aviation companies, so that the industry takes decisions towards the extensive use of these devices. The other aspect to be considered is the lack of agreed standards and certification of optical sensors for SHM in aircraft structures, which is an essential condition for the application of optical sensors for SHM on a large scale. FBGs represent the most mature and promising technology for this purpose and, even though they can be employed for other applications, they are mainly used for strain measurements. For this purpose, they present several advantages with respect to strain gauges, like their long-term stability, high multiplexing capability and suitability for being embedded into composite materials. Two other early-stage alternatives for strain measurement in aircraft structures are mentioned in this article. Regarding engines, optical sensors have provided excellent results for the evaluation of aeronautical turbines in ground tests, so the next challenge will be to apply them to aircraft engines in flight.
